# Seeing is believing: a breakthrough to visualize necrosomes in the tissue

**DOI:** 10.1038/s44321-024-00086-2

**Published:** 2024-06-10

**Authors:** Chongbo Yang, J Magarian Blander

**Affiliations:** 1 Jill Roberts Institute for Research in Inflammatory Bowel Disease, Weill Cornell Medicine, Cornell University, New York, NY USA; 2 Joan and Sanford I. Weill Department of Medicine, Weill Cornell Medicine, Cornell University, New York, NY USA; 3 Department of Microbiology and Immunology, Weill Cornell Medicine, Cornell University, New York, NY USA; 4 Sandra and Edward Meyer Cancer Center, Weill Cornell Medicine, Cornell University, New York, NY USA; 5 Immunology and Microbial Pathogenesis Programs, Weill Cornell Graduate School of Medical Sciences, Weill Cornell Medicine, Cornell University, New York, NY USA

**Keywords:** Autophagy & Cell Death, Methods & Resources

## Abstract

JM Blander and C Yang discuss a method for necroptosis detection in situ as reported by AL Samson, JM Murphy and colleagues, in this issue of *EMBO Mol Med*.

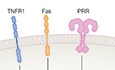

## Teaser

Detection of necroptosis in the tissue has been a long-standing roadblock in determining the disease states and pathological conditions associated with this inflammatory form of cell death. In this issue of EMBO Molecular Medicine, Chiou et al report a definitive method for necroptosis detection in situ (Chiou et al, 2024). The authors utilize this technical advance to unequivocally identify necroptosis lesions within the intestinal epithelium, and further reveal the simultaneous presence of distinct apoptotic and necroptotic lesions in human inflammatory bowel disease.

**Figure 1:**
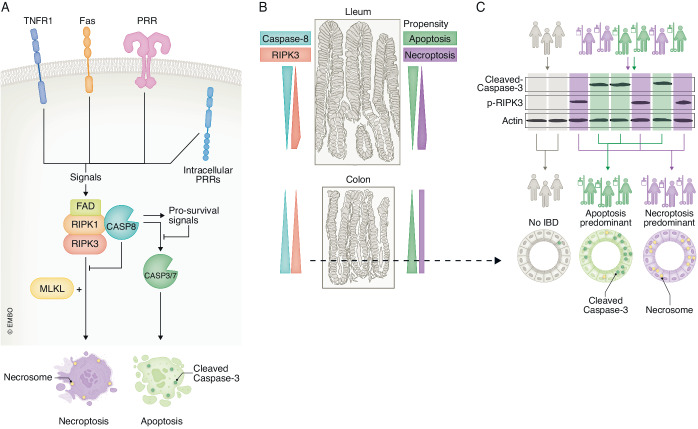
Detection of necrosome components reveals intricate cell death patterns in tissues and patients. (**A**) Inflammatory signaling from death receptors including pattern recognition receptors (PRRs) such as Toll-like receptor, Nod-like receptors, and other sensors are integrated by RIPK1 as a component of the Ripoptosome or Complex II downstream of TNF receptor signaling. The resultant protein complex plays a role in mediating pro-survival signals downstream of IKK/NF-κB and MAPK pathways, generating molecules that keep capsase-8 and RIPK1 in check. However, when this pro-survival response is perturbed, caspase-8 is cleaved into p19/20 and apoptosis effector caspases such as caspases 3 and 7 are activated, leading to apoptosis. Under certain conditions where caspase-8 is unfunctional, the constitutive inhibition of RIPK1 and RIPK3 to recruit MLKL is broken and a necrosome is generated, leading to necroptosis. (**B**) Distribution of caspase-8 shows an opposite trend in ileum versus colon. This distribution pattern might result in differential propensity of crypt intestinal epithelial cells to undergo apoptosis or necroptosis. (**C**) Colon samples from patients with inflammatory bowel disease (IBD) (green/purple) or healthy subjects (gray) are tested by immunoblotting to reveal the pre-dominant type of cell death in their IBD lesions. Patient colon sections are labeled for cleaved-caspase 3 and necrosome (indicated by punctate caspase 8 staining). Based on representative immunoblot results as shown, IBD patients are then categorized into either apoptosis (green) or necroptosis (purple) pre-dominant.

Tissue homeostasis involves constant cell turnover. Cell death is important to tissue homeostasis not only as the termination of unfunctional cells, but also as a source of instructive signals to the microenvironment. For instance, in the gut, the active propulsion of intestinal epithelial cells (IECs) favors apoptosis, whereby the released apoptotic bodies contribute to immune suppression and immune tolerance. On the other hand, necroptosis, a form of lytic cell death executed by the necrosome, releases necroptotic cellular debris and sends danger signals to the neighborhood, eliciting an inflammatory response in the tissue (Blander, 2018; Patankar and Becker, 2020). Thus, programmed cell death is driven by distinct programs in different situations, releasing specific repertoires of cellular components with distinct immunological consequences. Interestingly, the assembly of necrosomes is inhibited by caspase 8, a critical mediator of apoptosis (Clucas and Meier, 2023; Pasparakis and Vandenabeele, 2015). Therefore, necroptosis could be seen as a backup mechanism for failed apoptosis; the hallmark mechanism of homeostatic cell death (Fig. 1A).

Despite its unique position in the cell death kingdom, the relevance of necroptosis in inflammatory pathologies has been unfortunately difficult to confirm in vivo (Blander, 2018; Pasparakis and Vandenabeele, 2015). Until now, genetic defects that theoretically promote necroptosis have been consistently reported to be positively associated with inflammatory bowel disease (IBD), arthritis, and other autoimmunity in human and mouse models (Clucas and Meier, 2023; Mifflin et al, 2020). Nevertheless, a method for in situ detection of necroptosis using a definitive molecular marker in the tissue had been missing, and most studies have relied on subsidiary markers (e.g., TUNEL or electron microscopy), pharmacological inhibition or genetic knockout of critical necroptosis signaling molecules to prove the relevance of necroptosis in vivo, which either lacks specificity and/or availability in practice (Pasparakis and Vandenabeele, 2015). Moreover, the advantage of in situ assays is their ability to reveal not only the presence but also the distribution of necroptosis in the tissue, which is essential for understanding how necroptosis contributes to disease. As such, a standardized protocol to detect necroptosis in situ in the tissue has become an urgent issue that hinders progress of the entire field.

Chiou et al approached such a problem with solid and graceful work. Instead of targeting the technically more challenging detection of phosphorylated-RIPK3 and MLKL, the authors focused on the detection of components of the necrosome (including RIPK1, RIPK3, and MLKL) together with caspase-8. The authors first carried out a systemic screen to establish and optimize staining conditions for each marker with orthogonal variations in antibody clonality and concentration, blocking buffer components, antigen retrieval pH and timing in mouse Formalin Fixed Paraffin Embedded samples. Tissue from genetic knockout mice of corresponding staining target was used as the negative control, and signal to noise ratio was calculated iteratively for each condition, yielding optimized staining protocols for caspase 8, RIPK1, and RIPK3. The authors then applied these protocols in different tissues and observed a highly restricted pattern of RIPK3 expression across tissues, showing that RIPK3 is abundantly expressed in IECs, spleen, and Kupffer cells, but hardly detectable in heart muscle, kidney epithelial or brain. Such findings echoed public transcriptomic and proteomic datasets of various sources (Consortium et al, 2018; Geiger et al, 2013; Moor et al, 2018). As the propensity of cells to undergo necroptosis correlates to their expression of necroptotic machinery molecules (Pasparakis and Vandenabeele, 2015), the authors observations suggest that necroptosis probably plays a bigger role in the gut and spleen compared to kidney and heart. The authors’ staining further revealed intricate distribution of these critical molecules within the tissue. For instance, the authors showed that in the ileum, caspase-8 is more abundant towards the tips of villi, while in the large intestine, it is higher towards the crypts. On the other hand, RIPK3 expression is highest in regions next to the crypt in both the small and large intestine. Therefore, in the crypt, the ileum has a higher RIPK3/caspase-8 ratio compared to colon, which suggests that under inflammatory challenges, necroptosis would likely take place mostly near the crypt of ileum, while colon may be more resistant to necroptosis such that necroptosis may not show a strong pattern of enrichment in crypts (Fig. 1B). Such a deduction strategy could be applied to other tissues of interest, such as the spleen and liver.

The higher expression of necroptotic machinery in the gut and other barrier tissues could be due to higher inflammation baseline or the constitutive tissue-specific epigenomic modules. To address this possibility, the authors further studied the changes in necroptosis machinery distribution under the impact of TNF-α mediated acute inflammation, microbiota depletion by oral antibiotics, or vaccination with a model antigen. While robust increases in RIPK3 expression occurred at the site immune responses, microbiota depletion did not abolish the abundant RIPK3 expression in the gut, suggesting both inflammation and tissue imprinting play critical roles in regulating the distribution of necroptosis components.

The authors then proceeded with a similar methodology to obtain optimal protocols for detecting necroptosis components in human tissue. Interestingly, characteristic punctate staining patterns (Samson et al, 2021; Zhang et al, 2020) absent in apoptotic cells, inflamed cells, or untreated cells invariantly appeared in cells undergoing necroptosis when stained for necrosome components as well as for caspase-8, which allowed for in situ evaluation of necroptosis. In particular, the authors’ observations of the clustered caspase-8 pattern was unexpected and may reflect possible lingering of unfunctional caspase-8 on activating necrosomes in human tissue or a previously unknown mechanism that elicits caspase-8 clustering during necroptosis. Applying this finding, the authors were able to characterize necroptosis in situ in human IBD lesions. Although the relevance of necroptosis in intestinal inflammation is proven in genetic mouse models of RIPK3 and MLKL deficiency, such an approach is not feasible in humans. Moreover, the intertwined regulation of apoptosis and necroptosis (Clucas and Meier, 2023; Pasparakis and Vandenabeele, 2015) also make it difficult to manipulate one branch without altering the other. As a consequence, direct evidence to address the relevance of IEC necroptosis in human IBD, as well as the most basic and intriguing question of whether necroptosis is more damaging than apoptosis, has been missing (Blander, 2018; Pasparakis and Vandenabeele, 2015). To provide handles to these questions, the authors first evaluated the types of cell deaths in IBD lesions by immunoblotting and observed in different patients two types of IBD lesions, which are either dominated by apoptosis or necroptosis, as indicated by the extent of caspase cleavage versus RIPK3/MLKL phosphorylation. Excitingly, the type of cell death defined by immunoblotting correlates with the prevalence of necrosome in the crypt detected by immunohistochemistry for caspase-8 (Fig. 1C). This experiment not only validated the practice of punctate caspase-8 staining as the indicator of necrosome formation in human tissue, but also confirmed that different human IBD lesions are dominated by different types of IEC death. The assay reported by the authors will likely be key to future investigations of the roles of necroptosis in human IBD and other diseases. For instance, patients can be followed to understand how the relative prevalence of necroptosis versus apoptosis in their intestinal epithelium correlates with their history, disease activity, response to treatment, and clinical outcome. Moreover, in-depth analysis of the distribution of necroptosis within the tissue may yield hidden mechanisms by which necroptosis and local inflammation interact.

As discussed above, clearly the work of Chiou et al has successfully made a breakthrough of the long-standing bottleneck for necroptosis detection in the tissue, from which the field will much benefit in our future endeavor to get a clearer picture of whether and how necroptosis directly contributes to the make and break of barrier homeostasis.
